# Influence of Prostate Volume on the Incidence of Complications and Urinary Incontinence Following Thulium Fiber Laser Enucleation of the Prostate: Results from Multicenter, Real-world Experience of 2732 patients

**DOI:** 10.1016/j.euros.2024.03.004

**Published:** 2024-03-21

**Authors:** Daniele Castellani, Dmitry Enikeev, Mehmet Ilker Gokce, Vladislav Petov, Nariman Gadzhiev, Abhay Mahajan, Pankaj Nandkishore Maheshwari, Khi Yung Fong, Azimdjon N. Tursunkulov, Vigen Malkhasyan, Marek Zawadzki, Mario Sofer, Luigi Cormio, Gian Maria Busetto, Bhaskar Kumar Somani, Thomas R.W. Herrmann, Vineet Gauhar

**Affiliations:** a Urology Unit, Azienda Ospedaliero-Universitaria delle Marche, Università Politecnica delle Marche, Ancona, Italy; b Institute for Urology and Reproductive Health, Sechenov University, Moscow, Russian Federation; c Department of Urology, Medical University of Vienna, Vienna, Austria; d Department of Urology, Ankara University School of Medicine, Ankara, Turkey; e Department of Urology, Saint-Petersburg State University Hospital, Saint-Petersburg, Russia; f Sai Urology Hospital and Mahatma Gandhi Mission’s Medical College and Hospital, Aurangabad, India; g Urology Unit, Fortis Hospital Mulund, Mumbai, India; h Yong Loo Lin School of Medicine, National University of Singapore, Singapore; i Urology Division, AkfaMedline Hospital, Tashkent, Uzbekistan; j Urology Unit, A.I. Evdokimov Moscow State University of Medicine and Dentistry, Moscow, Russian Federation; k Urology Unit, St. Anna Hospital, Piaseczno, Poland; l Department of Urology, Tel-Aviv Sourasky Medical Center, Sackler Faculty of Medicine, Tel-Aviv University, Tel-Aviv, Israel; m Andrology and Urology Unit, Bonomo Teaching Hospital, Andria, Italy; n Department of Urology, Ospedali Riuniti di Foggia, University of Foggia, Foggia, Italy; o Department of Urology, University Hospitals Southampton, NHS Trust, Southampton, UK; p Department of Urology, Spital Thurgau AG, Kantonspital Frauenfeld, Frauenfeld, Switzerland; q Division of Urology, Department of Surgical Sciences, Stellenbosch University, Western Cape, South Africa; r Hannover Medical School, Hannover, Germany; s Department of Urology, Ng Teng Fong General Hospital, Singapore, Singapore

**Keywords:** Prostatic hyperplasia, Endoscopic enucleation of the prostate, Postoperative complications, Urinary incontinence, Thulium fiber laser

## Abstract

**Background:**

The use of the new thulium fiber laser in enucleation of the prostate (ThuFLEP) has been introduced recently.

**Objective:**

To evaluate complications and urinary incontinence (UI) after ThuFLEP in small and large prostate volume (PV).

**Design, setting, and participants:**

We retrospectively reviewed patients who underwent ThuFLEP in six centers (from January 2020 to January 2023). The exclusion criteria were concomitant lower urinary tract surgery, previous prostate/urethral surgery, prostate cancer, and pelvic radiotherapy.

**Outcome measurements and statistical analysis:**

Patients were divided into two groups: group 1: PV ≤80 ml; group 2: PV >80 ml. Univariable and multivariable logistic regression analyses were performed to evaluate the independent predictors of overall UI.

**Results and limitations:**

There were 1458 patients in group 1 and 1274 in group 2. There was no significant difference in age. The median PV was 60 (61-72) ml in group 1 and 100 (90-122) ml in group 2. En bloc enucleation was employed more in group 1, while the early apical release technique was used more in group 2. The rate of prolonged irrigation for hematuria, urinary tract infection, and acute urinary retention did not differ significantly. Blood transfusion rate was significantly higher in group 2 (0.5% vs 2.0%, *p* = 0.001). There was no significant difference in the overall UI rate (12.3% in group 1 vs 14.7% in group 2, *p* = 0.08). A multivariable regression analysis showed that preoperative postvoiding urine residual (odds ratio 1.004, 95% confidence interval 1.002-1.007, *p* < 0.01) was the only factor significantly associated with higher odds of UI. A limitation of this study was its retrospective nature.

**Conclusions:**

Complications and UI rates following ThuFLEP were similar in patients with a PV up to or larger than 80 ml except for the blood transfusion rate that was higher in the latter.

**Patient summary:**

In this study, we looked at outcomes after thulium fiber laser in enucleation of the prostate stratified by PV. We found that blood transfusion was higher in men with PV >80 ml, but urinary incontinence was similar.

## Take Home Message

Early and late complications following thulium fiber laser in enucleation of the prostate, except for the blood transfusion, was not significantly higher even in men with prostate volume >80 ml. Patients with high preoperative postvoiding urine residual had higher odds of having postoperative incontinence.

## 1 Introduction

Urologists were introduced to endoscopic enucleation of the prostate (EEP) in 1983, and as technology improved over the decades, it has gained global acceptance as a surgical approach for benign prostatic hyperplasia (BPH) management primarily with the advent of holmium laser and morcellators [1].

Increased rates of bleeding requiring transfusions, longer catheterization time, and hospital stay have historically been reported and considered a major concern after transurethral resection of the prostate (TURP) in men with large-volume glands [2]. The introduction of lasers in transurethral BPH surgery has reduced the incidence of bleeding complications and hospital stays dramatically [3].

The use of the new thulium fiber laser (TFL) in enucleation of the prostate (ThuFLEP) was first introduced in 2018 by Enikeev et al [4] who demonstrated that its efficacy was not inferior to TURP but had a better safety profile and higher decrease of prostate-specific antigen (PSA) value, showing its superiority in more complete removal of adenoma.

TFL beam has a wavelength of 1.940 nm that is near the water absorption peak [5]. The TFL energy pulse reduces only by 1.7% after traveling the distance of its optical penetration depth, and this in conjunction with its high water absorption guarantees a high energy supply to the prostatic tissue with a thin layer of carbonization, larger layers of cellular vacuolization, and a thermal-coagulation zone [6]. This provides excellent hemostasis in highly vascular tissue such as prostatic adenoma.

Currently, there are very few studies assessing the influence of prostate volume (PV) on ThuFLEP outcomes [7].

The primary aim of this study is to investigate the complication rates after ThuFLEP from a multicenter, real-world experience in different prostate sizes from small (up to 80 ml) to large glands (>80 ml). The secondary outcomes are to assess the incidence of and factors affecting postoperative urinary incontinence.

## 2 Patients and methods

We performed a retrospective analysis of all BPH patients who underwent ThuFLEP in six centers between January 2020 and January 2023. The inclusion criteria were acute urinary retention, lower urinary tract symptoms not responding to or worsening despite medical therapy, recurrent hematuria or urinary tract infections due to BPH, and bilateral hydronephrosis with renal impairment. The exclusion criteria were concomitant lower urinary tract surgery, previous prostate/urethral surgery, prostate cancer, and pelvic radiotherapy. Prostate cancer was ruled out before surgery in patients with elevated PSA or when clinically suspected by performing a prostate biopsy. At baseline, the following data were gathered: PV measured by transrectal ultrasonography, age, presence of a preoperative indwelling catheter, International Prostate Symptom Score (IPSS) with quality of life (QoL) item, PSA, postvoid residual urine (PVR), and maximum flow rate (Qmax) at uroflowmetry. Ten surgeons with previous experience of at least 200 ThuFLEP cases were involved in all procedures. Patients on oral anticoagulants were stopped or switched to low molecular weight heparin if deemed necessary in preparation for surgery and resumed as per each center's discretion, while single antiplatelet agents were maintained. All patients received antibiotic prophylaxis following local protocols. Enucleation was performed with a 26 Ch continuous-flow laser resectoscope, using either Urolase SP 60W (IPG Photonics, Oxford, MA, USA) or Fiber Dust (Quanta System, Milan, Italy). Morcellation was performed in all cases with Piranha (Richard Wolf, Knittlingen, Germany) or JAWS (Hawk, Minitech, China) morcellators. A 20 or 22 Ch bladder catheter was placed in the bladder after the procedure's completion, and continuous irrigation was maintained until the urine became clear. Enucleation time was considered as the time from the beginning of the enucleation until the start of morcellation. Surgical time encompassed the period from cystoscopy to catheter placement. Follow-up visits were scheduled at 1 and 3 mo after surgery in outpatient clinics, with subsequent appointments being determined based on individual cases.

Patients were divided into two groups based on the PV. PV threshold was chosen according to the European Association of Urology guidelines [8]. Group 1 included patients who had a PV up to 80 ml; group 2 consisted of patients with a PV above 80 ml. Complications were divided into early (within 30 d of surgery) and delayed complications. Early complications were classified according to the modified Clavien-Dindo classification. Urinary incontinence was defined as any complaint of urine leak according to patient reports and classified into the following: (1) stress urinary incontinence: involuntary loss of urine on effort or physical exertion, or on sneezing or coughing; (2) urge incontinence (UI): involuntary loss of urine associated with urgency; and (3) mixed urinary incontinence: both stress and urgency urinary incontinence [9]. The duration of incontinence was categorized into three groups according to the time between catheter removal and when patients reported that their incontinence had stopped: <1, 1-3, and 3 mo. The maximum length of follow-up was 1 yr.

Institutional board review approval was obtained by the leading center (Asian Institute of Nephrology and Urology, AINU #11/2022). The remaining centers had approvals from their institutional boards. All patients signed an informed consent form to collect their deidentified data.

### 2.1 Statistical analysis

Continuous variables are reported as median and interquartile range. Categorical variables are reported as absolute frequency and percentage. The chi-square test was employed to assess the difference between groups for categorical parameters and the Kruskal-Wallis test was used for continuous variables. A univariable analysis was performed to evaluate the factors associated with overall postoperative incontinence. Significant prognostic variables in a univariate analysis were entered into a multivariable generalized linear regression model to assess their significance as independent predictors. Predictors were described using odds ratios (ORs), 95% confidence intervals (CIs), and *p* values. A two-tailed *p* value of <0.05 was considered significant. All statistical tests were performed using R Statistical language, version 4.3.0 (R Foundation for Statistical Computing, Vienna, Austria).

## 3 Results

During the study period, 2732 patients met the inclusion criteria and were included in the analysis. There were 1458 patients in group 1 and 1274 in group 2. Table 1 shows patient baseline characteristics. There was no significant difference in age. The median PV was 60 (61-72) ml in group 1 and 100 (90-122) ml in group 2. There were a significantly higher proportion of patients with a preoperative indwelling catheter for acute urinary retention in group 2 (12.9% vs 5.8%, *p* < 0.001). There was no difference in preoperative QoL, while the median IPSS and Qmax were significantly higher in group 2, but this difference was not clinically meaningful. Preoperative PVR was significantly higher in group 2 (90 [66-100] vs 70 [50-80] ml, *p* < 0.001).

**Table 1:** Baseline characteristics of all patients and according to prostate volume

	Group 1 PV ≤80 ml (*N* = 1458)	Group 2 PV >80 ml (*N* = 1274)	*p* value
Age (yr), median (IQR)	67 (61, 72)	67 (61, 72)	0.33
Prostate volume (ml), median (IQR)	60 (50, 70)	100 (90, 122)	**<0.001**
Preoperative indwelling catheter, *n* (%)	85 (5.8)	164 (12.9)	**<0.001**
Preoperative IPSS, median (IQR)	23 (21, 25)	23 (21, 26)	**<0.01**
Preoperative QoL, median (IQR)	4.0 (4.0, 5.0)	4.0 (3.0, 5.0)	0.64
Preoperative Qmax (ml/s), median (IQR)	8.6 (7.0, 11)	8.5 (7.0, 10.7)	**0.02**
Preoperative PVR, median (IQR)	70 (50, 80)	90 (66, 100)	**<0.001**

IPSS = International Prostate Symptoms Score; IQR = interquartile range; PV = prostate volume; PVR = post-voiding residual of urine; Qmax = maximum flow rate; QoL = quality of life. Bold value stands for signifacnt p value.

Table 2 shows intraoperative characteristics. En bloc enucleation was employed more in group 1. Yet, there was a significantly higher use of the early apical release technique in group 2 (35.7% vs 26.5%, *p* < 0.001). Enucleation, morcellation, and total surgical time did not differ significantly between groups. The median postoperative catheter time was 2 (1.0-2.0) d in the whole series with no difference among the groups (2.0 [1.0, 3.0] d in group 1 vs 2.0 [1.0, 3.0] d in group 2, *p* = 0.52).

**Table 2:** Intraoperative characteristics of all patients and according to prostate volume

	Group 1 PV ≤80 ml (*N* = 1458)	Group 2 PV >80 ml (*N* = 1274)	*p* value
Patients on antiplatelets during surgery, *n* (%)	23 (1.6)	20 (1.5)	0.8
Patients on LMWH during surgery, *n* (%)	35 (2.4)	31 (2.1)	0.32
Enucleation type, *n* (%)			**<0.001**
3 lobes	16 (1.1)	88 (6.9)	
2 lobes	1133 (77.7)	999 (78.4)	
En bloc	309 (21.2)	187 (14.7)	
Early apical release, *n* (%)	386 (26.5)	455 (35.7)	**<0.001**
Total operation time, median (IQR))	65 (50, 90)	79 (60, 105.5)	**<0.001**
Enucleation time, median (IQR))	54 (40, 70)	70 (50, 95)	**<0.001**
Morcellation time, median (IQR))	20 (15, 30)	25 (15, 35)	**<0.001**
Spinal anesthesia, *n* (%)	1350 (92.6)	803 (63.1)	**<0.001**

IQR = interquartile range; LMWH= low molecular weight heparin; PV = prostate volume. Bold value stands for signifacnt p value.

Table 3 shows early and delayed complications and incontinence rates. The most frequent early complication in both groups was prolonged irrigation for hematuria (Clavien 2; 6.1% in group 1 vs 5.2% in group 2, *p* = 0.53), followed by urinary tract infection (Clavien 2; 3.5% in group 1 vs 3.8% in group 2, *p* = 0.70) and acute urinary retention (Clavien 2; 3.6% in group 1 vs 3.1% in group 2, *p* = 0.53). Blood transfusion rate was significantly higher in group 2 (Clavien 2; 0.5% vs 2.0%, *p* < 0.01), but there was no significant difference in surgical hemostasis for delayed bleeding within 30 d of surgery (0.6% in group 1 vs 1.3% in group 2, *p* = 0.12). The overall incidence of delayed complications was low and similar in both groups, with urethral stenosis requiring dilatation being the most common complication (1.4% in group 1 vs 1.2% in group 2, *p* = 0.78). Only one patient in group 2 required a reintervention for BPH.

**Table 3:** Postoperative outcomes and complications, and urinary incontinence rate

	Group 1 PV ≤80 ml (*N* = 1458)	Group 2 PV >80 ml (*N* = 1274)	*p* value
30-d complications, *n* (%)	218 (15.0)	218 (17.1)	0.14
Acute urinary retention requiring a catheter (Clavien 2)	52 (3.6)	39 (3.1)	0.53
Prolonged irrigation for hematuria (Clavien 2)	89 (6.1)	66 (5.2)	0.34
Blood transfusion (Clavien 2)	7 (0.5)	25 (2.0)	**0.001**
Postoperative bleeding requiring surgical hemostasis (Clavien 3)	9 (0.6)	16 (1.3)	0.12
Urinary tract infection (Clavien 2)	51 (3.5)	49 (3.8)	0.70
Sepsis (Clavien 4)	0 (0.0)	3 (0.2)	0.20
Secondary morcellation (Clavien 3)	10 (0.7)	14 (1.1)	0.34
Ureteral office injury requiring stenting (Clavien 3)	5 (0.3)	3 (0.2)	0.87
Cardiovascular complications (Clavien 2)	1 (0.1)	1 (0.1)	0.99
30-d readmission, *n* (%)	25 (2.0)	17 (1.5)	0.47
Overall postoperative incontinence, *n* (%)	180 (12.3)	187 (14.7)	0.08
Relation to time of follow-up for overall incontinence, *n* (%)	*N* = 93	*N* = 158	**<0.001**
<1	34 (36.6)	82 (51.9)	
1-3 mo	55 (59.1)	50 (31.6)	
>3 mo	4 (4.3)	26 (16.5)	
Type of incontinence, *n* (%)	*N* = 90	*N* = 158	0.9
Urge	25 (27.8)	49 (31.0)	6
Stress	47 (52.2)	71 (44.9)	
Mixed	18 (20.0)	38 (24.1)	
Relation to time of follow-up for each type of incontinence, *n* (%)	*N* = 90	*N* = 158	0.96
Urge			
<1 mo	9 (9.7)	28 (17.8)	
1-3 mo	15 (19.7)	12 (7.6)	
>3 mo	1 (1.1)	8 (5.1)	
Stress			
<1 mo	18 (19.4)	38 (24.1)	
1-3 mo	15 (19.7)	25 (15.7)	
>3 mo	0 (0.0)	8 (5.0)	
Mixed			
<1 mo	7 (7.5)	15 (9.5)	
1-3 mo	8 (10.5)	13 (8.2)	
>3 mo	3 (3.2)	10 (6.3)	
Delayed complications, *n* (%)			
Urethral stenosis requiring dilation only	20 (1.4)	15 (1.2)	0.78
Urethral stenosis requiring urethrotomy	2 (0.1)	8 (0.6)	0.07
Bladder neck stenosis requiring incision	9 (0.6)	13 (1.0)	0.34
Redo surgery for benign prostatic hyperplasia	0 (0.0)	1 (0.1)	0.95

PV = prostate volume. Bold value stands for signifacnt p value.

Regarding overall postoperative incontinence, there was no significant difference among the groups regarding incidence (12.3% in group 1 vs 14.7% in group 2, *p* = 0.08). Yet, the type of incontinence was also similar. Interestingly, the rates of patients with <1 and >3 mo UI were significantly higher in group 2.

At univariable analysis, preoperative indwelling catheter, preoperative PVR, no early apical release, and total operations time were the factors significantly associated with higher odds of having postoperative incontinence, but only preoperative PVR (OR 1.004, 95% CI 1.002-1.007, *p* < 0.01) was a significant factor at the multivariable analysis (Table 4).

**Table 4:** Univariable and multivariable analyses of factors associated with overall urinary incontinence

	Univariable analysis			Multivariable analysis		
	OR	95% CI	*p* value	OR	95% CI	*p* value
Age	1.005	0.991-1.02	0.50	-	-	
Prostate volume >80 ml (ref. ≤80 ml)	1.221	0.98-1.522	0.07	-	-	
Preoperative indwelling catheter	1.766	1.259-2.438	**<0.01**	0.742	0.230-2.265	0.60
Preoperative IPSS	1.021	0.998-1.047	0.08	-	-	
Preoperative Qmax	0.943	0.907-0.981	**<0.01**	0.978	0.925-1.033	0.42
Preoperative PVR	1.004	1.002-1.005	**<0.001**	1.004	1.002-1.007	**<0.01**
Enucleation type (ref. 3 lobes)						
2 lobes	0.407	0.258-0.662	**<0.001**	0.37	0.120-1.170	0.08
En bloc	0.788	0.483-1.32	0.35	0.525	0.174-1.617	0.25
No early apical release (ref. early apical release)	1.484	1.179-1.862	**<0.01**	1.493	0.491-4.458	0.47
Total operation time	1.005	1.002-1.008	**<0.001**	0.999	0.994 - 1.004	0.80

CI = confidence interval; IPSS = International Prostate Symptoms Score; OR = odds ratio; PVR = postvoiding residual of urine; Qmax = maximum flow rate; ref. = reference. Bold value stands for signifacnt p value.

## 4 Discussion

Despite ThuFLEP being the new “kid on the block” EEP procedure, it is advancing beyond both TURP and open simple prostatectomy, having demonstrated a lower rate of bleeding and shorter catheterization time and hospital stay [10], [11]. ThuFLEP has also shown a similar safety profile and functional outcomes to high-power holmium laser enucleation of the prostate [12], [13].

In this study, we assessed the incidence of complication and incontinence rates following ThuFLEP for clinical BPH, comparing patients with a PV of ≤80 ml versus those with a PV above 80 ml. Our study pointed out three important findings.

First of all, we found that the transfusion rate was low in both groups and we hypothesize that this can be attributed partly to the excellent coagulation ability of TFL. TFL has been shown in preclinical studies on soft tissues to have effective cutting proprieties with a high potential for hemostasis due to a low reduction of energy pulse during traveling and a high absorption coefficient in water (114 cm^−1^) [14]. Indeed, both the quasicontinuous and the superpulsed TFL showed excellent coagulation on a nonfrozen porcine kidney with a marked coagulation zone of 0.6 ± 0.1 and 0.4 ± 0.1 mm, respectively, at 70 W [15]. Despite this suitability for hemostasis, group 2 patients experienced a significantly higher blood transfusion rate even if no other reported complications related to bleeding, such as the need for intraoperative diathermy or bladder injury during morcellation, occurred in our series. Despite this difference, surgical hemostasis for delayed bleeding was similar between the groups. This result was in line with the study of Petov et al [7], who showed that surgical revision for clot retention did not differ in patients with a PV of <80 ml (0.6%%) as compared with those with larger volume glands (1.0%). However, we admit that we are not able to provide data on the use of intraoperative diathermy because data were collected retrospectively.

Another key observation in our study was the low rate of overall bladder neck stenosis (1.6%) within 1 yr of ThuFLEP. The etiology of bladder neck stenosis following BPH surgery is still not well understood, but the scar hypertrophy due to a prolonged inflammatory phase with poor bladder neck restoration is one of the hypotheses [16]. TFL has a low penetration depth and limited impact on soft tissues with minimal (quasicontinuous mode) or no (superpulsed mode) carbonization due to its high water absorption [14]. Therefore, the physical proprieties of TFL can partially explain our lower rate of bladder neck stenosis of ThuFLEP as compared with EEP performed with thulium:YAG (2.99%) [17] and holmium laser (3.2%) [18]. Conversely, the overall rate of urethral stenosis was quite high in our series (3.3%) as compared with the pooled incidence after EEP (1.7%) [19]. The etiology of urethral stenosis following transurethral surgery for BPH is also poorly understood, with energy employed, scope size, surgical time, and long pre- and postoperative catheterization time being among the hypothesized causes [19]. The cause of our urethral stenosis incidence was probably multifactorial, related to the scope size, higher rate of patients with a preoperative indwelling catheter, and recatheterization for acute retention. It would be interesting if, in future studies, smaller sheath sizes such as minimally invasive (22 Ch) and ultra slim (18.5 Ch) EEP using different energy have any influence on urethral stricture incidence [20].

The third finding in our study was the similar rate of overall postoperative incontinence between the groups. This result is not in line with the current concept of EEP that the larger the prostate, the higher the incontinence rate [21]. Petov et al [7] found a higher incidence of transient stress incontinence following ThuFLEP in men with a PV of ≥80 ml. This difference in results might partially be explained by the fact that patients with a PV of 80 ml were included in the small prostate group in our study and the large prostate group in Petov et al’s [7] study. However, the rate of incontinence lasting >3 mo was higher in group 2, and this could be explained by the common finding of a wider prostatic fossa that leads to the trapping of urine and subsequent easier leakage with stress maneuvers, and after detrusor contractions correlated to the change in bladder reaction to filling as an effect of altered feedback from the prostatic fossa [22]. Yet, we found that PVR was significantly associated with higher odds of postoperative incontinence, and there are some potential reasons for this. The sensory receptors of the bladder can become less sensitive when it is chronically distended. Postoperatively, this can lead to delayed or impaired signals to the brain [23], increasing the risk of leakage, particularly in those men suffering from persistent storage symptoms and de novo UI [24]. Chronic overdistension can also lead to reduced bladder compliance, which results in an increased likelihood of urgency and incontinence following relief of bladder outlet obstruction [25].

The present study has some limitations starting from the retrospective nature with its inherent bias. First, due to the retrospective analysis, we were not able to gather all minor complications. Second, we did not gather data on preoperative urinary incontinence, and thus, we were not able to assess the rate of de novo incontinence. Furthermore, the number of pads used and drug management of incontinence were not captured. Third, pre- and postoperative management of patients was not standardized, and this could add a bias. Fourth, the laser setting differed in each center as there is no consensus on the best laser setting for ThuFLEP, and we were unable to get a consensus for the same. Fifth, postoperative data on IPSS, Qmax, and PVR were not available for many patients, and this consequently hindered the ability to make a meaningful comparison between the groups. Finally, the outcomes of our study reflect the experiences of high-volume centers, and this may restrict the applicability of our findings to centers with less experience.

## 5 Conclusions

This study provides valuable insights into the outcomes of ThuFLEP in a large series of patients stratified by PV. While there were some baseline differences, the incidence of early and late complications, except for the blood transfusion rate, was not significantly higher even in men with PV >80 ml. Overall postoperative incontinence rates did not differ significantly between the groups. Patients with high preoperative PVR irrespective of the PV should be counseled regarding higher odds of having postoperative incontinence.

  ***Author contributions*:** Daniele Castellani had full access to all the data in the study and takes responsibility for the integrity of the data and the accuracy of the data analysis.

  *Study concept and design*: Gauhar, Castellani.

*Acquisition of data*: Gauhar, Gokce, Petov, Gadzhiev, Mahajan, Maheshwari, Tursunkulov, Malkhasyan, Zawadzki, Sofer, Cormio, Busetto.

*Analysis and interpretation of data*: Fong, Gauhar, Castellani.

*Drafting of the manuscript*: Gauhar, Castellani.

*Critical revision of the manuscript for important intellectual content*: Enikeev, Somani.

*Statistical analysis*: Fong.

*Obtaining funding*: None.

*Administrative, technical, or material support*: None.

*Supervision*: Herrmann.

*Other*: None.

  ***Financial disclosures*:** Daniele Castellani certifies that all conflicts of interest, including specific financial interests and relationships and affiliations relevant to the subject matter or materials discussed in the manuscript (eg, employment/affiliation, grants or funding, consultancies, honoraria, stock ownership or options, expert testimony, royalties, or patents filed, received, or pending), are the following: Thomas R.W. Herrmann is a consultant for, has received honoraria from, and is involved in research collaboration with Karl Storz. The remaining authors declare no conflict of interest.

  ***Funding/Support and role of the sponsor*:** None.
